# Issues in evaluating health websites in an Internet-based randomized controlled trial

**DOI:** 10.2196/jmir.4.3.e17

**Published:** 2002-10-17

**Authors:** Gunther Eysenbach

Critical, evidence-based evaluation of the effectiveness of information and communication technology should be one important component of eHealth [1]. In clinical medicine, randomized controlled trials (RCTs) are considered the "gold-standard" to assess the effectiveness of a treatment: next to systematic reviews, a well-conducted randomized trial can provide the strongest evidence for (or against) the effectiveness of an intervention. In a RCT, eligible patients are typically randomly assigned to receive either the new intervention or a control, for example, standard care or a placebo. Patients are then followed over a period of time and outcomes in both arms are measured and compared. Can we evaluate the effectiveness of a single health website in a similar way?

In this issue of the Journal, researchers at the Kaiser Permanente Center for Health Research present results of a study to develop and evaluate a web-based psychoeducational program designed to reduce depression [2]. This study represents one of the first randomized trials of a web-based mental health intervention ever conducted. A recent systematic review on comparative studies evaluating Internet-interventions stated a dearth of evidence in the field [3], and only one other recently published RCT known to us has evaluated a mental health intervention [4]. The results of the study published in this issue of the Journal are unexpectedly negative: the trial suggests that Internet-delivered mental health intervention may have no or limited treatment effects.

Two hundred and ninety-nine patients were randomly selected to receive either the online depression program in addition to usual care, or usual care alone. Previous studies evaluating traditional bibliotherapy (the treatment of depression with educational material) suggest that bibliotherapy is an effective intervention. Despite the fact that the Internet offers various possibilities to improve traditional educational and bibliotherapy material, the trial disappointingly did not result in any measurable effect in the overall sample. Although in an exploratory data analysis researchers found a small benefit for those participants who entered the study with lower levels of depression, these results need to be confirmed by subsequent studies. "We are at the very threshold of this burgeoning field, and we know very little about the circumstances and processes that will optimize the delivery and acceptance of these interventions", write Greg Clarke and colleagues. "There is no accumulated clinical lore about how to best provide Internet services; we are blazing this trail as we progress."

The study of Clarke and colleagues illustrates the possibilities and challenges of evaluating the impact of a health website on health outcomes in a web-based RCT.

Among the appealing factors is the fact that hundreds or thousands of people per day may frequent a health website, which gives plenty of sample size. Patients from all over the world can be recruited, enhancing the external validity (generalizability) of the results. No costly face-to-face interactions such as clinical examinations may be necessary if psychological outcomes are measured through self-administered electronic questionnaires. The administration of the intervention or the control, respectively, data collection and outcome measurement can be completely automatized. Not only does this make web-based RCTs very cheap, it minimizes biases, at least those introduced by human observers. Often an investigator's earliest opportunity to interact with the research subjects is when he opens the database to analyze the data.

On the other hand, there are significant challenges inherent in web-based randomized trials that don't exist in clinical trials studying drugs. Some of them are similar to challenges in educational interventions or surgical trials: For example, the trial cannot be conducted in a double-blind fashion as the patient always knows what intervention he receives.

But there are perhaps even more serious challenges. First, there is a considerable risk that the control group becomes "contaminated" by accessing a similar intervention from somewhere else on the web. This is particularly true if the intervention is "giving information" or an educational program that - in a similar way - can be easily found somewhere else on the web. Interventions such as smoking cessation programs can be easily found and used elsewhere on the web, threatening the ability to detect differences between the groups. Institutional review boards may require investigators to describe their intervention in detail before patients consent to participate. Participants who are randomized into the control group may be disappointed that they are not getting the intervention and may search the web for a comparable intervention, using it without the knowledge of the investigator. To minimize this bias the only option is to reduce the amount of information about the intervention given to participants, which may be ethically problematic. One should also ask participants in the control group whether they used similar interventions elsewhere on the web, or even monitor their use of other websites directly by using client-side proxy software.

Apart from the problem of ensuring that the control group actually stays a control group, we are facing the opposite problem in the intervention group: How do we assure that the intervention group is actually using the intervention? The relative ease of enrolling participants to a web-based trial seems to come at the cost of a high probability to lose them again - as many as about half of the patients [3; 4] may be lost to follow-up. In an intention-to-treat analysis such high drop-out rates greatly affect the ability to detect small differences between the groups. Even those who fill in the follow-up questionnaire (i.e. not dropped-out) may not actually have used the intervention, emphasizing the importance of asking about the frequency of use (or measuring it directly through log-files) and conducting a dose-response analysis. One may also have to think about employing novel techniques to reduce drop-outs. For example, preceding the actual trial one may employ a run-in period, where users are required to return to the website several times prior to enrollment and randomization. Only returning users will eventually be randomized into the intervention or control group.

The bias introduced by these issues is typically a "bias towards the null", i.e. through these methodological difficulties a trial may fail to show a small effect of an intervention, through the "noise" introduced. Despite these issues and despite alternative possibilities to evaluate a website (surveys, log-file analysis, before-after trials, and interrupted time series) the RCT remains the gold standard and we are eagerly looking forward to see more of these trials.
